# American Medical Association

**Published:** 1885-06

**Authors:** 


### AMERICAN MEDICAL ASSOCIATION.

### SECTION OF DENTAL AND ORAL SURGERY.

REPORTED FOR THE INDEPENDENT PRACTITIONER BY MRS. M. W. J.

The sessions of the American Medical Association for the year 1885, were held in the different halls of Tulane University, New Orleans, several sections also occupying rooms in Grunewald Hall, in the same square.

The section on Oral and Dental Surgery met in Grunewald Hall, at 3.30 P. M., Tuesday, April 28.

In the absence of Dr. W. W. Allport, Chicago, Ill., Chairman, and Dr. E. C. Briggs, Boston, Mass., Secretary, the meeting was called to order by Dr. John S. Marshall, who announced that the absence of Dr. Allport was due to the enforced order of his physician, as he was just recovering from protracted illness. To supply the vacancies, Jacob L. Williams, M. D., D. D. S., of Boston, Mass., was elected Chairman, and John S. Marshall, M. D., D. D. S., Chicago, Ill., Secretary. Among the members of the profession present were Dr. J. Mott Smith, Honolulu, Hawaiian Commissioner to the World's Exposition; Dr. A. E. Baldwin, Chicago; Oscar J. Coskery, M. D., Baltimore, and Drs. J. Knapp, G. J. Friedrichs,

J. R. Walker, and L. A. Thurber, of New Orleans. The local attendance was small, no notification having been given of the meetings of the Dental Section. An invitation was extended by the chair to local dentists to participate in the discussions, as guests of the American Medical Association.

The reading of the minutes of the last meeting was dispensed with, as they had already been published.

Dr. John S. Marshall then read a paper entitled "Cocaine in Dental Surgery." Hydrochlorate of Cocaine, like all new remedies which promise to mitigate the sufferings of mankind, was hailed with enthusiasm by the dental surgeon, on the record of its wondrous efficacy in Ophthalmology. It has, however, as a rule, proved a disappointment as an obtunder of sensitive dentine. Now that enthusiasm has waned, the wonderful effects at first attributed to it in this line, would appear rather the effect of imagination than clinical facts. New forms of cocaine, more recently introduced, seem to promise better results.

Dr. Marshall then proceeded to give the record of a series of cases treated—ten with the Hydrochlorate or Muriate, ten with the Oleate, and ten with the Citrate of Cocaine. The results were briefly as follows: The Hydrochlorate, or Muriate, proved to be almost valueless, and the two, four, ten, and twenty per cent solutions without any marked difference in value, perhaps because the dense tissues absorb so slowly that a sufficient quantity cannot be taken up.

In case No. 1, the result was so satisfactory that it was tried in nine successive cases, of which the details were given, with the following results :

1. Very satisfactory.
2. No diminution of sensitiveness.
3. Ditto.
4. Exceedingly painful.
5. No abatement.
6. Very slight diminution.
7. Considerable mitigation.
8. A little relief. Hot air blast gives still greater.
9. Hot air alone as beneficial as hot air and cocaine combined.
10. No perceptible benefit.

The next ten cases that presented, were treated with either the usual five per cent Oleate as found in the market, or the normal

Oleate, which is forty-eight to fifty per cent. The results were as follows:

1. After ten minutes, hurt very badly.

2. The gums lost all sensation; the tooth was sensitive, but not so much so as was anticipated.

3. Normal Oleate; no effect.

4. Ditto.

5. Ditto.

6. Five per cent, no effect; with hot air, a slight abatement; (the citrate was a perfect success.)

7. Five per cent; very slight effect.

8. (Band for artificial crown.) The gum was perfectly anæsthetized; no effect on dentine.

9. (Pyorrhœa.) Five per ccnt; the root was scraped with but little pain.

10. Tooth normally sensitive. Two applications diminished this somewhat, but only partially.

The next ten cases were treated with the Citrate of Cocaine, manufactured by Merck, and recommended by him as an obtundent of sensitive dentine. It was recommended in a letter published in the *Journal of the American Medical Association*, Dec. 20, 1884. None was to be obtained in this country, but McKesson & Robbins kindly prepared a sample. $[(C_{17} H_{21} NO_4)_2 \ H_3 C_6 H_5 O_7.]$

Mr. Thos. Whitfield, druggist, made it into pills of one-quarter grain each, using as an excipient gum tragacanth dissolved in glycerine. All debris was removed from the tooth, and the cavity was washed with tepid water. The rubber dam was then applied, and, dividing a pill into four parts, one or two pieces were placed in the cavity and covered with a pledget of cotton saturated with tepid water. This dissolves and spreads the remedy over the surface of the cavity. In five minutes a second application may be made, if necessary.

The following is the result of this treatment in ten successive cases:

1. After ten minutes, excavation was made without pain. The effect lasted one hour.

2. A perfect success.

3. The dentine was sensivive to heat and cold, also to acids or sweets. The least touch gave exquisite pain. The citrate was painful for twenty minutes, but after a second and third application, excavation became endurable.

(I had before tried the twenty per cent muriate solution without effect.)

4. A complete success.

5. Hypersensitive; removed the decay without pain.

6. A failure. (I afterward succeeded with oil of cloves and one-eighth grain of morphia sulphate, in five minutes.)

7. One-sixteenth of a grain was used. There was severe pain after five minutes. The second application of one-sixteenth of a grain was effectual.

8. A perfect success.

9. But little pain.

10. One application completely obtunded sensitiveness.

I have had many severe cases since, and in nearly every instance it has proved all that could be desired. Whether the results are due to this special form, or to the greater concentration of the active principle, I do not know. It acts more promptly, and with greater efficacy, whether in sensitive dentine, on exposed pulps, or on mucous membrane, than either of the other forms. It causes more or less pain when applied, the effect being similar to that produced by bibulous paper or spunk, in a cavity, or to the use of chloride of zinc. The effect passes off in from two to five minutes. It is perhaps caused by the rapid absorption of fluids from the tubuli. Although there are some unaccountable cases of utter failure, this record strongly hints that the perfect local anæsthetic has been found at last.

The paper elicited a lengthy discussion, which was opened by Dr. G. J. Friedrichs, of New Orleans. Having been out of practice for over two months, owing to a serious accident, he had not tried the Citrate. He had entirely failed with the Muriate and the Oleate. He could not agree with Dr. Marshall as to the alleged cause of pain in its application. The object of the hot air blast was to absorb the moisture from the tubuli, and thus to *prevent* pain.

This was the object proposed in the use of absolute alcohol, or Naboli, (which is alcohol and tannic acid).

*Dr. J. R. Walker*, New Orleans—Has experimented only with a four per cent solution of the Muriate. The results have been variable. In some cases it has obtunded sensitiveness to a great degree, so that patients have expressed the highest satisfaction. It is valuable also in lancing the gums previous to extraction, but the general results of his own experience, added to that of others, lead him to conclude that it is not what we need.

*Dr. J. Mott Smith*, Honolulu—A stranger here, I have listened to the remarks with great interest. If one thing is more especially desirable than another, it is a local anæsthetic which can be applied quickly, that shall render an operation easy, and prevent pain to the patient. From what he had seen of its effects on the eye, as used by a celebrated oculist in Albany, N. Y., he would expect Cocaine to be invaluable to the dentist. He had not heard its merits discussed before, as he had but recently arrived from the Sandwich Islands. He believes that the proper form for use in dental surgery will yet be found. Arsenic is a sure remedy for an exposed pulp, but it is too dangerous to place in inexperienced hands, or for use by the average dentist.

*Dr. Baldwin*, Chicago—Has only tried the Muriate, and his experience has been identical with that of Dr. Marshall. In one case, where the muriate had failed entirely, wishing to apply chloroform on a pledget of cotton, he accidentally got hold of the sulphuric ether bottle. He applied the cotton, saturated with ether, to the gum, where he supposed the end of the root of the superior left lateral incisor to be located. It was very painful at first, but after a few moments no more pain was experienced from the sensitive dentine. The patient did not know what had been applied, but there was certainly a lessened sensitiveness while excavating. In using arsenic, one one-hundredth grain is just as effectual as a grain, and a great deal safer. To neutralize the effects upon the soft tissues of any contact with an expressed surplus, I make an application of dialysed iron to the gum. The variation observed in the effects of Cocaine is perhaps due to different conditions in the circulatory and nervous system of the patient, rather than to the drug itself. If the pulp is in a sluggish, semi-devitalized condition, the result of chronic irritation, this application, or any other owing its effect to absorption and contact with neural structure, will be less effective than in a vigorous patient, or in healthy tissues. To judge of a series of experiments, we need information as to the condition both of the patient and of the tooth. I hope Cocaine will prove to be what we need.

*Dr. Friedrichs*—If a healthy pulp is exposed, it is not sensitive. In the case of a young boy, twelve years old, who fell and split a tooth, exposing the pulps completely, I touched it with my excavator repeatedly. It was not in the least painful, and yet it was fully exposed. There was no more feeling in it than in the gum. It is only when the pulp is irritated and diseased that it becomes painful. Then Cocaine should act. Into freely exposed, healthy pulps, you can drive a splinter of wood and cause no pain.

*Dr. Williams*—Have you seen many such cases ?

*Dr. Friedrichs*—Yes, a great many. I often cut off the crown and expose the pulp, then drive in an orange-wood or hickory splinter, dipped in carbolic acid. It gives no pain, because there is no feeling in a freely exposed, healthy pulp. The results from Cocaine are variable, even in the same mouth. In one case I used the four per cent hydrochlorate in sensitive cavities on one side of the mouth, and excavated and filled without difficulty. On the opposite side of the same mouth, at the same time, under the same conditions, it produced no effect whatever. Why should it be a success on one side and not on the other?

*Dr. Marshall*—How old was your solution ? Was there any fungus in it ?

*Dr. Friedrichs*—I had it one week. It was procured from Keppler, but I do not know if he prepared it himself. We must remember that the action of Coaine is only local. In operations in the eye,where the affection is deep-seated, chloroform is still required. We must remember, too, that there is no mucous membrane in tooth structure.

*Dr. Baldwin*—I am very much surprised to hear that a healthy pulp is not sensitive; that we can drill into a tooth and cause no pain. I find it quite the contrary sometimes. I can remember several cases in which the pulp was very sensitive; in fact, I never knew of an instance in which it was otherwise. Dr. Friedrichs labors under a misapprehension. I intended to say that the application was effective or not, depending upon the condition of the nerve-tissue of the pulp, or the mucous membrane of the gum (not of the tooth). If there were no terminal nerve-fibres, we should have no sensitive dentine by communication with the pulp, not through the gum, but through the nerve-fibres in the canaliculi. The action of an obtundent is due to the destruction or diminution of the conductive power of nerve tissues.

*Dr. Walker*—I have never touched a live pulp without finding it highly sensitive, though it may be so benumbed or paralyzed by a sudden blow as to be insensible to pain at the time, and this was probably the case with the tooth of the boy cited by Dr. Friedrichs. A dead pulp, of course, is not sensitive, but I believe it is generally admitted that the live pulp of a tooth, when fully exposed in a cavity, whether accidentally or otherwise, is the most exquisitely sensitive structure in the human body. I feel greatly indebted to Dr. Marshall for the record of his experience with the Citrate of Cocaine, and shall take the earliest opportunity to try it.

*Dr. L. A. Thurber*, New Orleans—My experience with the Muriate of Cocaine is quite contrary to that of Dr. Friedrichs. I could not dispense with it in my office. I think failures are often due to allowing the cocaine to be washed away by the fluids of the mouth. A pledget of cotton, saturated with cocaine, is allowed to be freely washed in the fluids of the mouth, and the remedy is so diluted that it has no effect. I first apply the rubber dam, and then dry the cavity, and never fail of success where all fluids are excluded. It is valuable in extracting, when used hypodermically, but the application is painful, and it is also difficult to go through the foramen, which must be done to render it effective. In opening an abscess, or for the removal of a pulp where devitalization is absolutely necessary, it saves more pain than any other agent I have ever used.

*Dr. Coskery*, Baltimore—In a case of very severe pain for twelve hours, one single application of hydrochlorate was a perfect success.

*Dr. Williams*—Experiences with this, as with any other material, are variable. I have known cases in which chloric-ether was a perfect success, and others again where it had no effect.

The application of tannin is often very painful, and aqua-calcis will often relieve the pain. In fact, aqua-calcis itself often relieves sensitive dentine. The effect of either the two per cent., the four per cent., or the thirty per cent. muriate solution, varies in the same way.

*Dr. J. R. Walker* (to Dr. Marshall)—Did I understand that your success was greater in decalcified teeth?

*Dr. Marshall*—It would seem to vary with the density of the teeth. When the structure is very dense, the effect is less. I would like to ask Dr. Thurber if he uses only the hydrochlorate?

*Dr. Thurber*—That is the only form that I use. It is very important to keep the cavity perfectly dry.

*Dr. Marshall*—I have used it both with and without the rubber dam, in forty or fifty cases, but did not find that it affected the result. I have tried drying with spunk, and with the hot-air blast, but the hydrochlorate fails, with or without these adjuncts. Have failed utterly with two, four, ten and twenty per cent. solutions. I know my preparation was perfectly fresh and good when received, though in the course of three weeks a fungus growth will form, when it becomes perfectly valueless.

*Dr. Baldwin*—Alcohol or glycerine will preserve it.

*Dr. Marshall*—The oleate was not designed for the teeth. It is intended for use on external surfaces—to anæsthetize the surface of the body, where the hydrochlorate has no effect.

*Dr. Thurber* (to Dr. Marshall)—How long did you wait for the effect of the hydrochlorate ?

*Dr. Marshall*—From ten minutes to an hour, before giving it up.

*Dr. Thurber*—Did you fail in very clean teeth ?

*Dr. Marshall*—I failed in teeth of every character.

*Dr. Thurber*—It is successful every time, in my hands, and especially with children.

*Dr. Marshall*—Your experience is very different from mine. I will add that the citrate is a new form, prepared by Merck, and recommended by him for this purpose. McKesson & Robbins promise to keep it for us. It should be made up into pills of one-eighth gr. each, and each pill divided into two or four pieces, as required by the cavity. In this shape it is always ready for use, and does not deteriorate. In aqueous solution it spoils in three days. Its effect on the tongue is complete in one minute; hydrochlorate requires three minutes; but the effect of the citrate is not so profound, though more rapid. At present it costs eighty cents per grain; the price of the muriate is about sixty cents.

The discussion was closed.

Dr. Jacob L. Williams, M. D., D. D. S., of Boston, read a paper entitled "The Alternation of Rest with Effort."

The discussion of this paper was deferred until the next session.

Adjourned.

